# PU.1 Regulates Cathepsin S Expression in Large Yellow Croaker (*Larimichthys crocea*) Macrophages

**DOI:** 10.3389/fimmu.2021.819029

**Published:** 2022-01-05

**Authors:** Xiang-Yang Zhang, Xinyue Zhuo, Jie Cheng, Xiaohong Wang, Kexin Liang, Xinhua Chen

**Affiliations:** ^1^ Key Laboratory of Marine Biotechnology of Fujian Province, Institute of Oceanology, College of Animal Sciences (College of Bee Science), Fujian Agriculture and Forestry University, Fuzhou, China; ^2^ Southern Marine Science and Engineering Guangdong Laboratory (Zhuhai), Zhuhai, China

**Keywords:** Large yellow croaker (*Larmichthys crocea*), macrophage, PU.1, cathepsin S, IFN-γ

## Abstract

Different morphologies have been detected in teleost macrophages. In this study, two macrophage cell lines were sub-cloned from a large yellow croaker head kidney cell line, LYCK. One type of sub-cloned cells was fusiform but the other was round, named LYC-FM and LYC-RM cells respectively, based on their morphologies. Both types showed the characteristics of macrophages, including expression of macrophage-specific marker genes, possession of phagocytic and bactericidal activities, and production of reactive oxygen species (ROS) and nitric oxide (NO). The transcription factor PU.1, crucial for the development of macrophages in mammals, was found to exist in two transcripts, PU.1a and PU.1b, in large yellow croaker, and constitutively expressed in LYC-FM and LYC-RM cells. The expression levels of PU.1a and PU.1b could be upregulated by recombinant large yellow croaker IFN-γ protein (r*Lc*IFN-γ). Further studies showed that both PU.1a and PU.1b increased the expression of cathepsin S (CTSS) by binding to different E26−transformation−specific (Ets) motifs of the CTSS promoter. Additionally, we demonstrated that all three domains of PU.1a and PU.1b were essential for initiating CTSS expression by truncated mutation experiments. Our results therefore provide the first evidence that teleost PU.1 has a role in regulating the expression of CTSS.

## Introduction

Macrophages, the first line of innate responders controlling and organizing host defense against pathogens, are found across all vertebrate species and reside in virtually all animal tissues (1, 2). They are essential for maintaining the balance of the immune system, repairing injured tissue, and eliminating invading pathogens (3). Macrophages are able to discriminate self from non-self, sense tissue damage, and recognize invading pathogens (3). When pathogens invade, macrophages can rapidly kill them by engulfment and production of reactive oxygen intermediates and nitric oxide synthase-2 (NOS2)-dependent reactive nitrogen intermediates, phagolysosomal acidification, and restriction of nutrient availability (2).

Primary macrophages have been isolated in several bony fish, including rainbow trout (*Oncorhynchus mykiss*) (4), Atlantic salmon (*Salmo salar*) (4), Atlantic cod (*Gadus morhua* L.) (5), goldfish (*Carassius auratus* L.) (6), common carp (*Cyprinus carpio*) (7), hogchoker (*Tminectes maculatus*) (8), dab (*Limanda limanda*, L.) (9), rohu (*Labeo rohita*) (10, 11), half smooth tongue sole (*Cynoglossus semilaevis*) (12), and large yellow croaker (*Larmichthys crocea*) (13). Meanwhile, macrophage cell lines have been established in rainbow trout (14), goldfish (15), catla (*Catla catla*) (16), rohu (17), and large yellow croaker (18). It is worth noting that primary macrophages or macrophage cell lines in bony fish exhibit diverse morphologies, including round, fusiform, and spreading shapes. For example, both goldfish primary macrophages and macrophage cell lines are round (6, 15), whereas a fusiform morphology is found either in primary macrophages or in macrophage cell lines in rohu (10). Furthermore, rainbow trout macrophages have round and spreading morphologies in both primary macrophages and macrophage cell lines (14, 19).

PU.1, encoded by the Spi1 gene, is a member of the E26−transformation−specific (Ets) family of transcription factors. It is selectively expressed in hematopoietic tissues and essential for the development and maturation of myeloid (granulocytes and monocytes/macrophages) and lymphoid cells (20). It has been demonstrated that PU.1 is abundantly expressed in terminally-differentiated macrophages and dendritic cells (DC), and is necessary for the development of macrophages (21). In addition, some cytokine receptors involved in the development of monocytes, such as macrophage colony-stimulating factor receptor (M-CSFR), granulocyte colony-stimulating factor receptor (G-CSFR), granulocyte macrophage colony-stimulating factor receptor (GM-CSFR), and interleukin-7 receptor (IL-7R), can be regulated by PU.1 (20, 22). Moreover, PU.1 can control the proliferation of macrophages by upregulating M-CSFR (23). PU.1 also serves as an important factor in macrophage maturation by regulating the expression levels of genes that are involved in cell differentiation (24).

PU.1 contains three functional domains: a transactivation domain, a PEST domain, and an ETS domain. The transactivation domain is required for the M-CSF-dependent proliferation of macrophages (23). The PEST domain is rich in proline (P), glutamic acid (E), serine (S) and threonine (T) residues, and is involved in controlling protein stability and proteolytic degradation (25). The ETS domain is responsible for the binding of PU.1 to the Ets motif [(G/A)GAA] within the promoter of its target genes (26).

Cathepsin S (CTSS) is a member of the cysteine proteases that mediate the proteolysis of endocytosed polypeptides (27). It is mainly expressed in antigen presenting cells (APCs), including B cells, dendritic cells, and macrophages. The expression level of CTSS can be upregulated by IFN-γ through PU.1 binding to its promoter (28, 29). It has been demonstrated that cells constitutively expressing CTSS also exhibit high expression levels of PU.1 (28). In our previous study, we demonstrated that CTSS was constitutively expressed in primary kidney macrophage (PKM) and LYCK cells in the large yellow croaker (*Larimichthys crocea*) (30). Whether these cells expressed PU.1 is still unclear. Thus, it is necessary to explore the relationship between PU.1 and CTSS in this species. This will be helpful for clarifying the function of PU.1 in bony fish macrophages.

In this study, two types of cells were isolated from LYCK cells *via* a limiting dilution assay. One type displayed a fusiform morphology, and the other type had a round morphology; these were named LYC-FM and LYC-RM cells, respectively. Both types showed the characteristics of macrophages, including the expression of macrophage-specific marker genes, possession of phagocytic and bactericidal activities, and production of reactive oxygen species (ROS) and nitric oxide (NO). Thereafter, two transcripts of PU.1 gene (PU.1a and PU.1b) were cloned, and both were detected in LYC-FM and LYC-RM cells. Both PU.1a and PU.1b could upregulate CTSS expression by binding to the different Ets motifs of its promoter. Additionally, we demonstrated that each of the three domains of PU.1a and PU.1b were essential for induction of CTSS expression by using truncated mutation experiments. Our results provide the first evidence for an immunoregulatory role of teleost PU.1 on CTSS expression.

## Materials and Methods

### Establishment of Macrophage Cell Lines

To establish macrophage cell lines from LYCK cells, a limiting dilution assay was used. Briefly, a series of double dilutions was carried out to dilute the original 5×10^4^ cells into 16 concentration gradients, and then cells in every concentration gradient were seeded in 96-well plates (Guangzhou Jet Bio-Filtration Co., Ltd.) with 200 μl complete L-15 medium (L-15 medium [Gibco] supplemented with 2.2 g/L NaCl, 0.66 g/L HEPES, 100 U/mL penicillin, 100 μg/mL streptomycin and 10% fetal bovine serum [Gibco]) and cultured at 28°C. Ten days later, single or nearly single clones were selected, and the medium was replaced with fresh complete L-15 medium. Cells in selected wells at about 80% density were harvested, and a new series of double dilutions was performed as described above. Single clones in the second dilution plate were selected and cultured, and cell morphology was observed under a microscope (Nikon). Subsequently, macrophage-specific marker genes reported in murine animals and other teleost fish were examined by real-time PCR in LYC-FM and LYC-RM cells as well as in PKM. The methods for PKM isolation were the same as our previously study (31). Briefly, head kidney tissue from large yellow croaker was passed through 70 μm nylon mesh and suspended in L-15 medium (Gibco), and cell suspensions were layer onto a 34/51% discontinuous Percoll (GE Healthcare) density gradient and centrifuged at 650 × *g* for 30 min. The band of leukocytes lying at the interface was collected and put on culture dish for 2 h at 28°C. After cell attachment, the attached macrophages were digested with trypsin (Gibco) and suspended in L-15 medium. The purity of primary kidney macrophages was identified based on Wright-Giemsa staining.

### Wright-Giemsa Staining

Wright-Giemsa staining was performed with a Wright-Giemsa Stain Kit (Nanjing Jiancheng Bioengineering Institute) according to the manufacturer’s instructions, with minor modifications. Briefly, 5×10^5^ cells were put on a slide, and about 100 μl solution R1 was added to the slide. After one minute, 200 μl of solution R2 was added for another five minutes’ reaction. Then, ultrapure water was added and mixed, followed by 30 s of staining. Finally, the stained cells were observed under a microscope (Nikon).

### Phagocytosis Assay

A total of 2×10^5^ cells in 500 μl L-15 incomplete medium (L-15 medium supplemented with 2.2 g/L NaCl, 0.66 g/L HEPES, 100 U/mL penicillin, and 100 μg/mL streptomycin) were added to 24-well plates (Guangzhou Jet Bio-Filtration Co., Ltd.) that were previously plated with fluorescent beads (FluoSpheres™ carboxylate, 1 μm, red, Life Technologies Corporation) by centrifugation at 2,500 × *g* for 5 min at a cell:bead ratio of 1:50. After incubation at 28°C for 10 h, non-ingested beads were removed by centrifugation (100 × *g* for 10 min at 4°C) over a cushion of 3% (weight/volume) BSA (Thermo Scientific) in PBS supplemented with 4.5% D-glucose (Sigma-Aldrich). After three washes, cells were suspended in 200 μl PBS, followed by observation under a fluorescence microscope (Nikon). Phagocytic activity and mean fluorescence intensity (MFI) were detected using a BD Accuri™ C6 Plus Flow Cytometer (BD Biosciences). Phagocytic activity was expressed as the percentage of cells that ingested beads.

### Bacterial Killing Assay

To detect the bacterial killing activity of isolated cells to *Pseudomonas plecoglossicida*, an *in vitro* bacterial killing assay was performed. The *P. plecoglossicida* used in this study was separated from affected large yellow croaker and were preserved in our laboratory, as described in a previous study (32). Macrophages were treated with long-phase *P. plecoglossicida* at a multiplicity of infection (MOI) of 50 and were incubated for 1 h, and non-engulfed *P. plecoglossicida* were removed by washing with sterile PBS. One group of engulfed cells was lysed in 1% Triton X-100 solution and plated onto tryptic soy agar plates (the uptake group). The other group of engulfed cells were incubated for another 1.5 h with complete L-15 medium supplemented with 100 μg/mL gentamicin (Amresco) (the killing group). The cells were collected, lysed in 1% Triton X-100 solution, and plated onto tryptic soy agar plates. Colonies of viable bacteria were counted after 18 h incubation at 28°C, and the survival rates of bacteria were represented as percentages of colonies in the killing group/colonies in the uptake group.

### Cellular ROS Activity

The level of ROS was measured with a 2,7-dichlorofluorescin diacetate (DCFH-DA) cellular ROS assay kit (Beyotime Biotechnology, China) according to the manufacturer’s instructions with some modifications. Isolated cells (10^5^ cells/well) in 96-well plates were stained with 100 μl diluted DCFH-DA for 30 min at 28°C in the dark. The DCF fluorescence intensity of 5000 cells was read using a fluorescence microplate reader (GloMax Discover, Promega) with an excitation wavelength of 475 nm and an emission wavelength of 500–550 nm. ROS activity was represented as DCF fluorescence intensity. To detect ROS activity after stimulation, 10^5^ cells/well in 96-well plates (Guangzhou Jet Bio-Filtration Co., Ltd.) were incubated with 1 μg/mL recombinant large yellow croaker IFN-γ protein (r*Lc*IFN-γ) or recombinant Trx protein (rTrx) (as a control) at 28°C for 6 h. Proteins used in this study were the same as in our previously study (31). Briefly, the DNA fragment encoding mature peptide of large yellow croaker IFN-γ was inserted into a pET32a (+) plasmid (Novangen) with a 6 × His tag. The resulting recombinant plasmid pET-IFN-γ was used to express recombinant large yellow croaker IFN-γ in *E. coli* BL21 Star (DE3) competent cells (Invitrogen). The r*Lc*IFN-γ was purified using a Ni-NTA Superflow resin (Qiagen) and preserved in PBS (pH = 7.4). After incubation, cells were stained, and the DCF fluorescence intensity of 5000 cells was read. Relative ROS activity was calculated as percentage of the value for the r*Lc*IFN-γ well/rTrx well.

### Nitric Oxide Assay

The level of nitric oxide (NO) was measured by the Griess assay using a nitric oxide detection kit (Beyotime Biotechnology, China) according to the manufacturer’s instructions. In the assay, 10^5^ cells were cultured in 96-well plates (Guangzhou Jet Bio-Filtration Co., Ltd.) under normal conditions; the supernatants were harvested, and NO concentrations were measured. Absorbance values at 540 nm were acquired with Infinite M Nano (Tecan), and NO concentration was determined using a sodium nitrite standard curve. To detect the level of NO after stimulation, 10^5^ cells/well in 96-well plates were cultured and treated with 1 μg/mL r*Lc*IFN-γ or rTrx protein (as a control) at 28°C. After 48 h, cell supernatants were collected, and NO concentrations were measured. Relative NO concentration was calculated as the percentage of the value for r*Lc*IFN-γ protein per well/rTrx protein per well.

### Cloning and Sequence Analysis of the Large Yellow Croaker PU.1 Gene

Based on the transcript sequences of PU.1 gene from the large yellow croaker genome database [ (33, 34); GenBank accession No. XM_010748147 and XM_010748148], primers PU1-F and -R (see **Table S1**) were designed for amplification of the open reading frame (ORF) of large yellow croaker PU.1 protein. The PCR products were cloned and sequenced at Sangon Biotech (Shanghai) Co., Ltd. Multiple sequence alignments of corresponding amino acid sequences of the obtained PU.1 was performed with ClustalW (Version 1.83) and shaded using the GeneDoc program. Protein identification was conducted using the Expert Protein Analysis System (http://www.expasy.org/tools/), and the signal peptide was predicted using the SignalP program (http://www.cbs.dtu.dk/services/SignalP/). Phylogenetic trees were constructed by the neighbor-joining (NJ) method using the MEGA program (version 5.05) and with 1000 bootstrap replicates.

### Expression Levels of Large Yellow Croaker PU.1 in Macrophages

To know the expression levels of PU.1 in macrophages under normal conditions, PKM, LYC-FM, or LYC-RM cells were collected. Total RNA was extracted using Eastep Super Total RNA Extraction Kit (Promega), and first-strand cDNA was synthesized from the total RNA by using an Eastep RT Master Mix (Promega). Quantitative real-time PCR (qRT-PCR) was performed with the gene-specific primers (see **Table S1**). The reactions were conducted in duplicate and each 10 μl reaction volume contained 2 μl cDNA template, 5 μl SYBR qPCR master mix (Vazyme Biotech Co., Ltd.), and 0.25 μl of each forward and reverse primer (10 μM). The amplification profile was analyzed using Quant Studio 5 (Applied Biosystems). Gene expression levels were normalized to that of β-actin.

### Plasmid Construction

The ORFs of PU.1a and PU.1b of large yellow croaker were cloned into the *EcoR*I site of pCMV-Flag-MAT-Tag1 (Sigma-Aldrich) using the primers PU1-F1/-R1. Primers PU1-F2/-R1, PU1-F1/-R2/-F3/-R1, and PU1-F1/-R3 were used to amplify the three PU.1 mutants, and then the mutants were cloned into the *Eco*RI site of pCMV-Flag-MAT-Tag1 (Sigma-Aldrich). For promoter activity analysis, the promoter fragment of the large yellow croaker CTSS gene was cloned into the *Kpn*I and *Hind*III sites of pGL3-Basic vector (Promega). Primers used for plasmid construction are listed in **Table S1**.

### Cell Transfection

EPC and LYC-FM cells were cultured at 28°C in L-15 medium supplemented with 15% FBS. HEK 293T cells were maintained at 37°C, 5.0% CO_2_ in DMEM medium supplemented with 10% FBS. For EPC cells transfection, the cells were seeded in 24-well plates overnight and transfected with about 1 μg plasmids suspended in 2 μl of FUGUN HD transfection reagent (Promega) and 100 μl of OPTI-MEM 1 Reduced Serum Medium (Invitrogen). For LYC-FM cells transfection, 5×10^6^ cells with 2 μg plasmids were transfected using a LONZA Nucleofector 2B Device according to the manufacturer’s instructions. Protein expression was measured by Western blot analysis as described previously (35). For the chromatin immunoprecipitation (ChIP) assay, HEK 293T cells seeded in 9-cm dishes were co-transfected with 5 μg Flag-tag plasmids and 5 μg relative promoter constructions. Cells were harvested after 48 h, and cell lysates were immunoprecipitated with anti-Flag-Agose beads (Beyotime Biotechnology, China). Then the immunoprecipitates were analyzed by semi-quantitative PCR.

### Luciferase Activity Assay

EPC cells were seeded in 24-well plates, and co-transfected with 500 ng luciferase reporter plasmids, 500 ng PU.1 plasmids, and 10 ng PRL-TK vector (Promega). Forty-eight hours after transfection, cells were harvested and luciferase activities were measured by a Dual-Luciferase Reporter Assay System (Promega) according to the manufacturer’s instructions. Luciferase activities were measured by GloMax Discover (Promega) and normalized to the Renilla luciferase activity. The means of three independent experiments were used for statistical analysis.

### Chromatin Immunoprecipitation Assay

The ChIP assay was performed using a ChIP Assay Kit (Beyotime Biotechnology, China) according to the manufacturer’s instructions with a minor modification. Briefly, HEK 293T cells were co-transfected with promoter plasmids and PU.1 plasmids, and 270 μl of 37% formaldehyde was added to 10 mL media for crosslinking of intracellular protein-DNA complexes. The cells were incubated for 10 min at room temperature, and then 0.125 M glycine was added to the medium to quench unreacted formaldehyde. The medium was discarded; the cells were washed twice with cold PBS, scraped into microfuge tubes, centrifuged at 4°C, and lysed with 1 mL of SDS Lysis Buffer. Cell lysates were disrupted by Vibra-Cell Ultrasonic Liquid Processors (Qsonica). After being centrifuged at 4°C for 10 min, 100 μl of sheared cross-linked chromatin was added into the tube containing 900 µl of Dilution Buffer. To remove protein-DNA complexes that may bind nonspecifically to agarose, 60 μl of Protein A+G Agarose/Salmon Sperm DNA was added. The mixtures were centrifuged, and 10 μl of the supernatant was collected as input, while the remaining supernatants were incubated with anti-Flag-Agarose (Beyotime Biotechnology, China) overnight at 4°C. Immunoprecipitated Flag-tagged protein-DNA complexes were washed with Low Salt Immune Complex Wash Buffer, High Salt Immune Complex Wash Buffer, LiCl Immune Complex Wash Buffer, and TE Buffer. Then, 100 µl of Elution Buffer was added to each tube containing the Flag-tagged protein-DNA complex for 15 min and the supernatants were collected. The collected immunoprecipitates and inputs were treated with 5 M NaCl at 65°C for 4–5 h, 1 µl RNase A at 37°C for 30 min, and a solution containing 4 µl 0.5 M EDTA, 8 µl 1 M Tris-HCl and 1 µl proteinase K was added and incubated at 45°C for 2 h. The DNA was purified using a PCR Purification Kit (Beyotime Biotechnology, China), and semi-quantitative PCR was used to analyze promoter DNA using specific primers (**Table S1**).

### Statistics Analysis

Statistical analysis was carried out by one-way analysis of variance (ANOVA) with Dunnett’s *post hoc* test (SPSS Statistics, Version 20). All experiments were repeated at least three times. A *p* value < 0.05 was considered statistically significant.

## Results

### Macrophage Cell Lines Establishment

**Figure 1:**
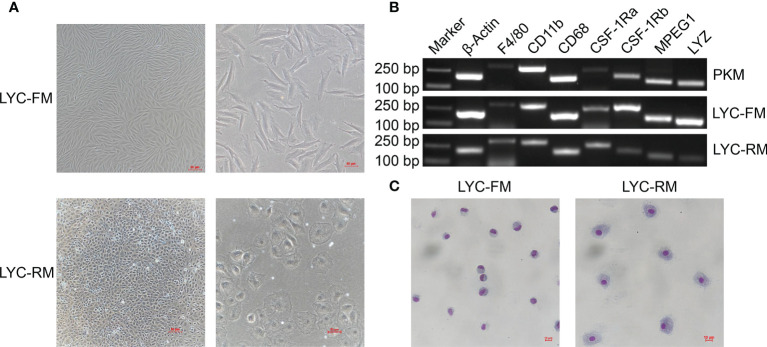
Morphologies and Wright-Giemsa staining of LYC-FM and LYC-RM cells. **(A)** LYC-FM and LYC-RM cells were observed under ordinary optics microscope. Left panel: original magnification ×10; right panel: original magnification ×20. **(B)** Macrophage-specific marker genes were detected in PKM, LYC-FM and LYC-RM cells by semi-quantitative PCR. **(C)** Wright-Giemsa staining of LYC-FM and LYC-RM cells. Cells were harvested, put on slide, and stained with Wright-Giemsa reagents. Then cell morphology was observed under ordinary optics microscope.

Using the limit dilution method, single cells were sub-cloned from LYCK cells for establishment of high purity cell lines. We screened eight single clonal wells and found two types of cells. As shown in **Figure 1A**, one cell line had fusiform morphology and the other type had round morphology. The fusiform cells were morphologically similar to the macrophages in rohu (17, 36) and catla (16), while the round cells were similar to the macrophages in goldfish (6, 15) and primary macrophages in large yellow croaker (13, 37). According to their shapes, these two sub-cloned cells were named LYC-FM and LYC-RM cells, respectively. Based on the similar morphologies to the macrophages observed in other teleost fish, we hypothesized that these two sub-cloned cells may be macrophages. Then, we tested macrophage-specific marker genes reported in murine animals and other teleost fish, including F4/80, CD11b, CD68, CSF-1R, MPEG1, and LYZ. As shown in **Figure 1B**, all detected macrophage marker genes were expressed in LYC-FM and LYC-RM cells. These results were consistent with those in PKM, indicating that the two cell lines sub-cloned here were macrophages. To further confirm the sub-cloned cell types, Wright-Giemsa staining was applied. Both LYC-FM and LYC-RM cells displayed characteristics associated with those of macrophages, with a claret-colored nucleus and blue cytoplasm (**Figure 1C**). The nuclei of LYC-FM cells showed a crescent-shaped morphology and were larger than those of LYC-RM cells. Compared with LYC-FM cells, LYC-RM cells were larger than in diameter, and contained a round nucleus and greater amount of cytoplasm. The morphology of LYC-RM cells was closer to that of primary macrophages in large yellow croaker as previously reported (31). All these results indicated that the sub-cloned single cell lines were the macrophages with different morphologies in large yellow croaker.

### Functions of Macrophage Cell Lines

**Figure 2:**
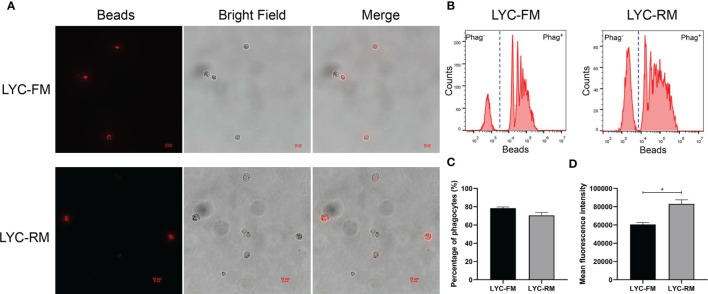
Phagocytosis by LYC-FM and LYC-RM cells with fluorescent latex beads. **(A)** Immunofluorescence microscopy of LYC-FM and LYC-RM cells incubated *in vitro* with 1 μm red fluorescent latex beads. **(B)** Phagocytosis of 1 μm red fluorescent latex beads by LYC-FM and LYC-RM cells. LYC-FM and LYC-RM cells were incubated *in vitro* with beads for 10 h, and then bead phagocytosis was thereafter measured by flow cytometry. Figure shows histograms of cell number versus fluorescence intensity representative of uptake activity. Increased peak fluorescence denotes more ingested fluorescent beads. Phag^-^, non-phagocytic cells; Phag^+^, phagocytic cells. **(C, D)** Percentage and mean fluorescence intensity of phagocytes in LYC-FM and LYC-RM cells. Data are representative of three independent experiments (mean ± SEM). (*p < 0.05).

To detect whether LYC-FM cells or LYC-RM cells had phagocytic activity, a fluorescent bead ingestion experiment was conducted. Internalization of beads by LYC-FM and LYC-RM cells was confirmed by immunofluorescence microscopy (**Figure 2A**). Thereafter, we analyzed the percentage and mean fluorescence intensity (MFI) of phagocytic cells. A higher percentage of phagocytosis was found within the LYC-FM cells (78.4% of the cells ingested beads) compared with LYC-RM cells (70.5% of the cells ingested beads) (**Figures 2B, C**). In contrast, the phagocytic capacity of LYC-RM cells was significantly higher than that of LYC-FM cells, since a higher MFI was found in LYC-RM cells (~83,000) compared with LYC-FM cells (~60,000) (**Figure 2D**).

**Figure 3:**
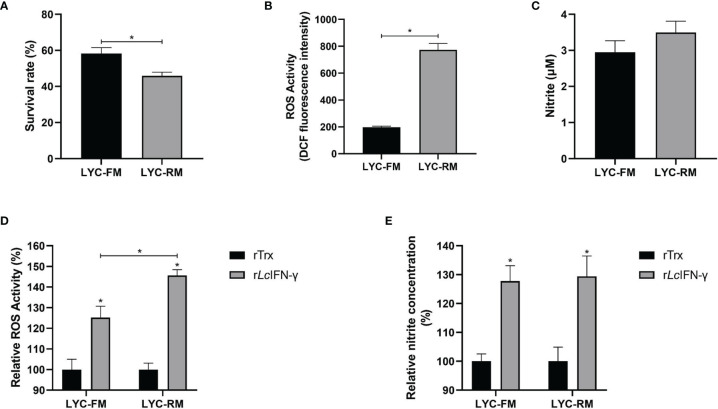
Functions of LYC-FM and LYC-RM cells. **(A)** Bacterial killing ability to *P. plecoglossicida.* LYC-FM or LYC-RM cells were treated with *P. plecoglossicida* at a MOI of 50 for 1 h alone (the uptake group) or additional 1.5 h (the killing group), and survival rates were presented as percentage of the colonies in the killing group/colonies in the uptake group. **(B)** ROS activity in LYC-FM and LYC-RM cells. Cells were harvested under normal conditions and stained with 2,7-dichlorofluorescin diacetate. The fluorescence intensities of the cells were read using a fluorescence microplate reader, and ROS activity was represented as DCF fluorescence intensity. **(C)** Nitrite oxide activity in LYC-FM and LYC-RM cells. Cells were cultured under normal condition and supernatants were harvested. NO concentrations were measured using the Griess reaction. **(D, E)** The r*Lc*IFN-γ protein induced the expression of ROS and NO. Data are representative of three independent experiments (mean ± SEM) (*p < 0.05).

To determine whether LYC-FM and LYC-RM cells had bactericidal activity, colonies of viable bacteria were counted after co-cultivation of *P. plecoglossicida* and cells. As shown in **Figure 3A**, both LYC-FM and LYC-RM cells had bactericidal activity, and LYC-RM cells had a stronger bactericidal activity than LYC-FM cells (54.1% vs. 41.8%). Fish macrophages can destroy the invading pathogens *via* generation of ROS such as superoxide and its metabolites (38, 39). We next detected ROS production in LYC-FM and LYC-RM cells under normal conditions. As shown in **Figure 3B**, the ROS activity in LYC-RM cells was significantly higher than that in LYC-FM, as a higher DCF fluorescence intensity was found in LYC-RM cells (~774) compared with LYC-FM cells (~197). NO is important for macrophages to kill bacteria, and r*Lc*IFN-γ can induce the release of NO in large yellow croaker primary macrophages (31). To investigate whether LYC-FM and LYC-RM cells produced NO, we next detected the NO levels in LYC-FM and LYC-RM cells under normal conditions. As shown in **Figure 3C**, both LYC-FM and LYC-RM cells could release NO under normal conditions, similar to the results observed in large yellow croaker primary macrophages (31). To determine whether ROS and NO production in LYC-FM and LYC-RM cells can be induced, changes in ROS and NO levels were analyzed in r*Lc*IFN-γ-treated LYC-FM and LYC-RM cells. As shown in **Figures 3D, E**, ROS and NO production in LYC-FM and LYC-RM cells could be enhanced by r*Lc*IFN-γ compared with the rTrx-treated cells, with a higher ROS level in LYC-RM cells than in LYC-FM cells.

### Molecular Characterization of Large Yellow Croaker PU.1a and PU.1b

**Figure 4:**
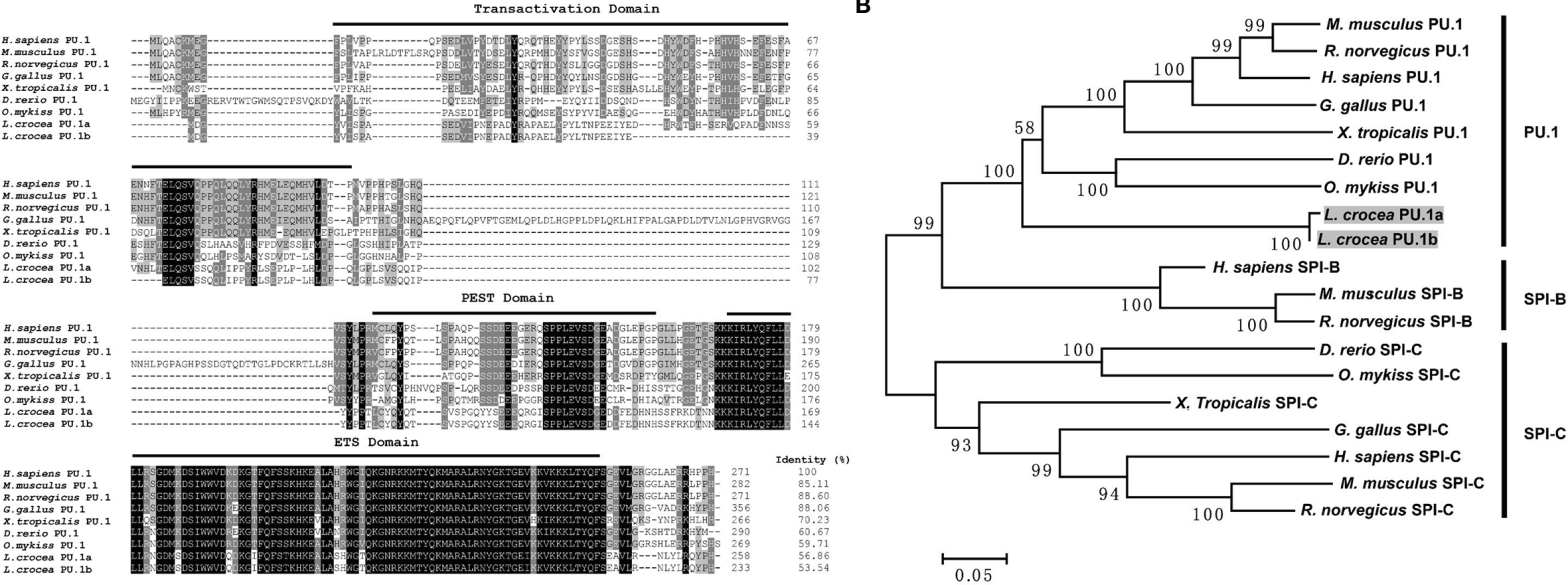
Multiple sequence alignments and phylogenetic tree of large yellow croaker PU.1a and PU.1b with selected PU.1 molecules. **(A)** Multiple sequence alignments of large yellow croaker PU.1a and PU.1b with selected vertebrate PU.1. The identical and similar residues from the aligned sequences are shaded black and gray respectively. **(B)** An unrooted phylogenetic tree of vertebrate PU.1. Percentage values shown for each node represented 1000 bootstrap replications. In the multiple alignment and phylogenetic tree construction, GenBank accession numbers are as follows: *H sapiens* PU.1, NP_001074016; *M. musculus* PU.1, NP_001365827; *R. norvegicus* PU.1, NP_001005892; *G gallus* PU.1, NP_001376298; *X. tropicalis* PU.1, NP_001139455; *D rerio* PU.1, NP_001315297; *O. mykiss* PU.1, NP_001117985; *L. crocea* PU.1a, XM_010748147; *L. crocea* PU.1b, XM_010748147; *H sapiens* SPI-B, NP_001230927; *M. musculus* SPI-B, NP_063919; *R. norvegicus* SPI-B, NP_001019457; *H sapiens* SPI-C, NP_689536; *M. musculus* SPI-C, NP_035591; *R. norvegicus* SPI-C, NP_001101550; *G gallus* SPI-C, XP_025011560; *X. tropicalis* SPI-C, XP_031753998; *D rerio* SPI-C, NP_001004621; *O. mykiss* SPI-C, XP_021432878.

Two transcripts of PU.1 gene were identified in large yellow croaker, and their ORFs were 777 and 702 bp, encoding 258 and 233 amino acids, respectively, tentatively named PU.1a and PU.1b. Both of them contained a transactivation domain, a PEST domain, and an ETS domain, based on SMART program prediction. PU.1a shared 56.86% identity to human PU.1, whereas PU.1b shared only 53.54% identity (**Figure 4A**). Phylogenetic analysis further indicated that all fish PU.1 molecules, including PU.1a and PU.1b, were grouped together and clustered with the PU.1 molecules from higher vertebrates, but were separated from SPI-B and SPI-C (**Figure 4B**), indicating that the PU.1a and PU.1b cloned here were two transcripts of PU.1.

### Expression Patterns of PU.1a and PU.1b in Macrophages

**Figure 5:**
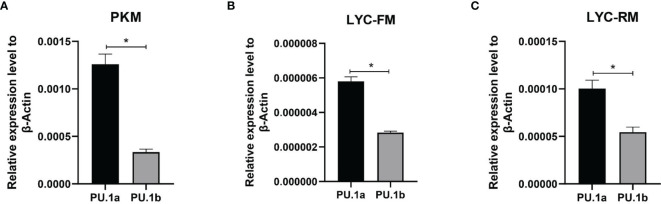
Expression patterns of large yellow croaker PU.1a and PU.1b in macrophages. The expression of PU.1a and PU.1b in PKM **(A)**, LYC-FM **(B)** and LYC-RM cells **(C)** were analyzed by qRT-PCR, and the expression level was normalized with that of β-actin. Data are representative of three independent experiments (mean ± SEM) (*p < 0.05).

The expression levels of PU.1a and PU.1b were examined in macrophages using qRT-PCR. To investigate the expression levels of PU.1a and PU.1b in macrophages, PKM, LYC-FM, and LYC-RM cells under normal condition were collected and their mRNA expression levels were detected. As shown in **Figure 5**, both PU.1a and PU.1b were constitutively expressed in PKM, LYC-FM, and LYC-RM cells, and the expression level of PU.1a was significantly higher than that of PU.1b.

### IFN-γ, PU.1a, and PU.1b Induced CTSS Expression

**Figure 6:**
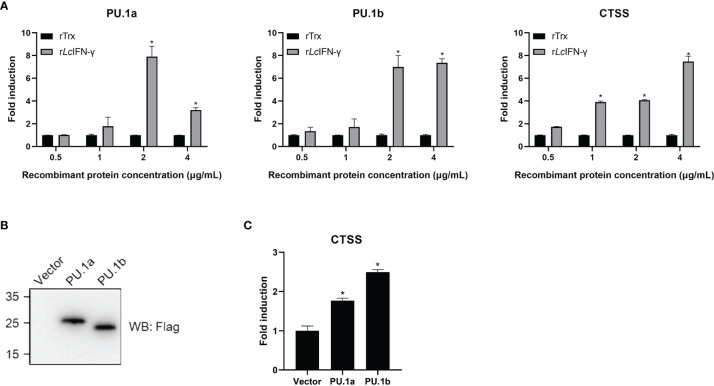
mRNA expression of the PU.1a, PU.1b and CTSS triggered by IFN-γ, PU.1a or PU.1b. **(A)** LYC-FM cells were treated with r*Lc*IFN-γ protein, cells were harvested at 48 h later. Then, total RNAs were extracted to examine the mRNA levels of PU.1a, PU.1b and CTSS through qRT-PCR. **(B, C)** LYC-FM cells were transfected with control vector, PU.1a or PU.1b respectively. Cells were harvested at 48 h later. Proteins of PU.1a and PU.1b were detected by Western blot analysis with anti-Flag antibody, and CTSS mRNA level induced by PU.1a or PU.1b were detected through qRT-PCR. Data of gene expression levels are representative of three independent experiments (mean ± SEM) (*p < 0.05).

In mammals, CTSS expression can be induced by IFN-γ through PU.1 (22). To investigate whether a similar mechanism is also present in large yellow croaker, the r*Lc*IFN-γ-treated LYC-FM cells were used as a model to analyze the expression changes of PU.1a, PU.1b, and CTSS. As shown in **Figure 6A**, the expression levels of PU.1a, PU.1b, and CTSS all were upregulated by the r*Lc*IFN-γ. Then, we explored whether PU.1a and PU.1b could induce the expression of CTSS. Because LYC-FM cells had a higher growth rate than LYC-RM cells, we used LYC-FM cells as a model to analyze expression changes of CTSS under overexpression of PU.1a or PU.1b. Western blot analysis showed that PU.1a and PU.1b could overexpressed in recombinant plasmid-transfected LYC-FM cells (**Figure 6B**). It was found that overexpression of both PU.1a and PU.1b could significantly increase the expression level of CTSS (at 48 h), and PU.1b was more potent than PU.1a in upregulating CTSS expression (**Figure 6C**).

### PU.1a and PU.1b Bind to the CTSS Promoter to Initiate Its Expression

**Figure 7:**
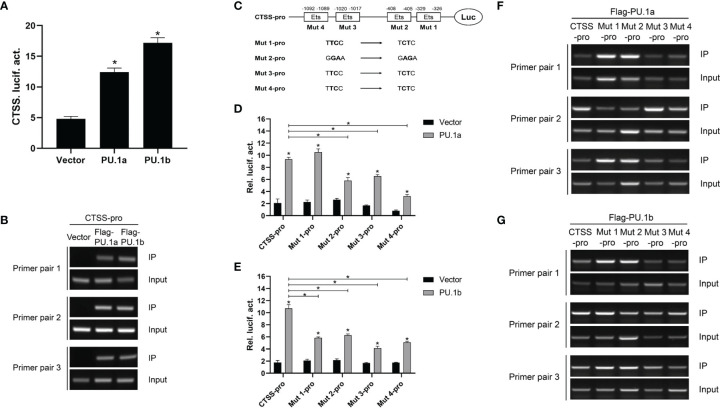
CTSS expression activated by PU.1a and PU.1b. **(A)** The CTSS promoter activities driven by PU.1a and PU.1b. EPC cells seeded in 24-well plate were co-transfected with 0.5 µg pCMV-Flag (control vector), Flag-PU.1a or Flag-PU.1b and 0.5 µg CTSS promoter plasmid. pRL-TK was used as an internal control. Promoter activities were monitored at 48 h after transfection. **(B)** The binding of PU.1a or PU.1b with CTSS promoter. HEK 293T cells were co-transfected with 5 µg pCMV-Flag (control vector), Flag-PU.1a or Flag-PU.1b and 5 µg CTSS promoter plasmid. After 48 h, cell lysates were immunoprecipitated with anti-Flag-Agarose beads. Then the DNA binding to CTSS promoter was checked by semi-quantitative PCR. The input was used as a control to quantify the DNA concentration. **(C)** Schematic representation of mutated CTSS promoter-driving luciferase constructs. **(D, E)** The function of Ets motifs on activation of CTSS. EPC cells were co-transfected with pCMV-Flag (control vector), Flag-PU.1a or Flag-PU.1b and the mutants of CTSS promoter plasmid. Promoter activities were monitored at 48 h after transfection. **(F, G)** The binding of PU.1a or PU.1b with Ets motifs of CTSS promoter. HEK 293T cells were co-transfected with pCMV-Flag (control vector), Flag-PU.1a or Flag-PU.1b and the mutants of CTSS promoter plasmid. Then the DNA binding to CTSS promoter was checked by ChIP assay. Data are representative of three independent experiments (mean ± SEM) (*p < 0.05).

The aforementioned results suggested that IFN-γ may induce the expression of CTSS *via* PU.1a and PU.1b, and thus it was necessary to determine whether these two transcription factors could activate the promoter of CTSS. As shown in **Figure 7A**, both PU.1a and PU.1b could activate the promoter activity of CTSS. To verify that PU.1a and PU.1b directly promoted CTSS expression, ChIP assays were performed. Empty vector, PU.1a or PU.1b-containing plasmids, and CTSS promoter were co-transfected into HEK 293T cells, and the results showed that both PU.1a and PU.1b could directly bind to the CTSS promoter (**Figure 7B**). The promoter of the large yellow croaker CTSS contains four putative Ets motifs (**Supplementary Figure S1**), which have been considered crucial for PU.1 binding in mammals (22). To clarify which Ets motif is required for binding of large yellow croaker PU.1a and PU.1b to CTSS promoter, we constructed four mutants (Mut 1-pro, 2-pro, 3-pro and 4-pro) of the CTSS promoter, with four Ets motifs mutated (**Figure 7C**). The activities of Mut 2-pro, 3-pro and 4-pro were significantly decreased after transfection with PU.1a or PU.1b, indicating that these Ets motifs were essential for the binding of CTSS promoter with PU.1a and PU.1b (**Figures 7D, E**). In addition, the first Ets motif was essential for binding of CTSS promoter with PU.1b but not for binding of CTSS promoter with PU.1a (**Figures 7D, E**). CTSS promoter activity could be significantly suppressed after mutation of these related Ets motifs, but the activities of these CTSS promoter mutants were significantly increased by PU.1a or PU.1b compared with those by empty vector (**Figures 7D, E**). These results indicated that a single Ets motif mutant could decrease but not completely inhibit CTSS promoter activity. The ChIP assays showed that both PU.1a and PU.1b could still bind to CTSS promoter with a single Ets motif mutation (**Figures 7F, G**). These results indicated that a single Ets motif mutation did not affect the direct binding of PU.1a or PU.1b to CTSS promoter.

**Figure 8:**
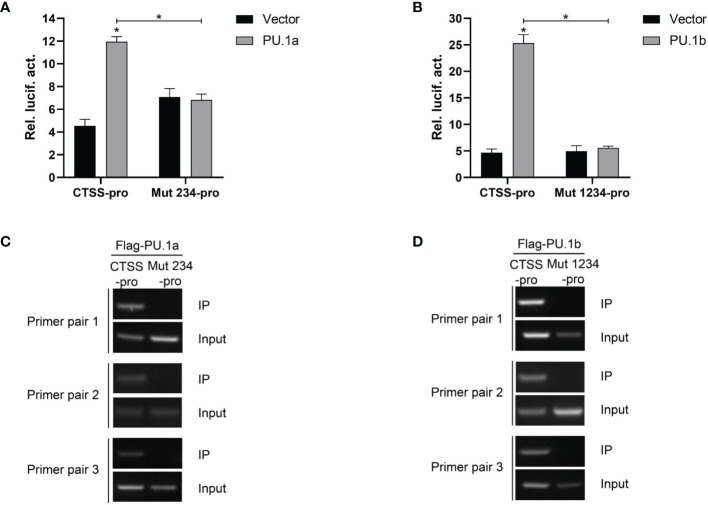
Mutations of Ets motifs attenuate CTSS activation and abrogate PU.1a or PU.1b binding to CTSS promoter. **(A, B)** Mutations of Ets motifs attenuate CTSS activation. **(C, D)** The binding of PU.1a or PU.1b with mutants of Ets motifs. Data are representative of three independent experiments (mean ± SEM) (*p < 0.05).

Thereafter, the last three or all four Ets motifs of the CTSS promoter were mutated, and the resulting mutants were named Mut 234-pro and Mut 1234-pro, respectively. The results showed that neither PU.1a nor PU.1b could activate Mut 234-pro or Mut 1234-pro activity compared with the empty vector (**Figures 8A, B**). The ChIP assay results also demonstrated that neither PU.1a nor PU.1b could directly bind to Mut 234 or Mut 1234 mutants of CTSS promoter (**Figures 8C, D**). These results indicated that the last three or all four Ets motifs in CTSS promoter were essential for PU.1a or PU.1b-induced promoter activity.

### All of Three Domains of PU.1a and PU.1b Are Required for Driving CTSS Promoter Activity

**Figure 9:**
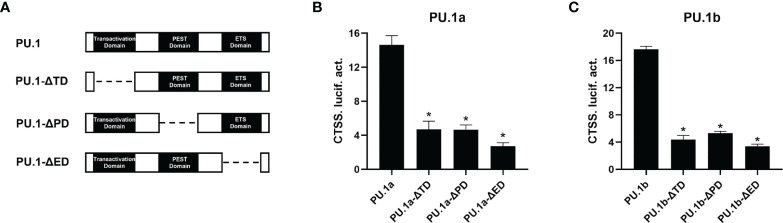
Effects of the different structural domains of PU.1a and PU.1b on CTSS promote. **(A)** Schematic representation of wild type PU.1a or PU.1b and their three mutants. **(B, C)** PU.1a or PU.1b mutants on CTSS promoter. HEK 293T cells were co-transfected with wild type or mutations of PU.1a or PU.1b and CTSS promoter, luciferase activities were monitored at 48 h after transfection. Data are representative of three independent experiments (mean ± SEM) (*p < 0.05).

To characterize the involvement of the functional domains of PU.1a and PU.1b in regulation of the promoter of CTSS, three functional domains, transactivation domain, PEST domain, or ETS domain, of PU.1a or PU.1b were mutated and truncated mutants, dubbed PU.1-ΔTD, PU.1-ΔPD, and PU.1-ΔED, were constructed (**Figure 9A**). Thereafter, the promoter activity of CTSS was detected. As shown in **Figures 9** **B, C**, the promoter activity of CTSS could not be activated by these mutants, indicating that all three domains of PU.1a and PU.1b are required for driving CTSS promoter activity.

## Discussion

Macrophages are the main effector cells of the innate immune response, and play critical roles in host defense and immune homeostasis. In fish, one of the obstacles in understanding the immune functions of macrophages is the unavailability of macrophage cell lines. In this study, two macrophage cell lines from large yellow croaker were established and characterized. Thereafter, we have used these two macrophage cell lines to investigate the functions of PU.1a and PU.1b in regulating the CTSS expression.

Primary macrophages have been isolated in bony fish, and macrophage cell lines have also been established in several species. In these primary macrophages or macrophage cell lines, different morphologies have been detected, including fusiform, round, and spreading shapes (6, 10, 14, 15, 17, 19). In large yellow croaker, primary macrophages show a round morphology, whereas macrophage cell lines are fusiform (13, 40). Here, another two macrophage cell lines were sub-cloned from an established large yellow croaker head kidney cell line, LYCK (**Figure 1A**). Macrophage marker genes have been detected in these two cell lines, including F4/80, CD11b, CD68, CSF-1Ra/b, MPEG1 and LYZ (**Figure 1B**). The marker genes of F4/80, CD11b and CD68 have been demonstrated to be expressed in murine macrophages (41), and CSF-1Ra/b, MPEG1 and LYZ genes have been detected in bony fish primary macrophages (42, 43). These results demonstrated that the selected single cell lines were macrophages. Wright-Giemsa staining is a typical method for detecting cell types, and bony fish macrophages have also been detected using this staining (42). The Wright-Giemsa staining results showed that two sub-cloned macrophage cell lines had claret-colored nuclei and blue cytoplasm (**Figure 1C**), consistent with macrophages in rainbow trout and zebrafish (*Danio rerio*) (42, 44). This further supported that both types of cells sub-cloned here were macrophages in large yellow croaker.

Phagocytosis is a fundamental defense mechanism in macrophages that plays a dominant role in defense against invading pathogenic bacteria in fish (6, 45, 46). In the present study, both LYC-FM and LYC-RM cells were demonstrated to engulf fluorescent latex beads, and the phagocytic capacity of LYC-RM cells was significantly higher than that of LYC-FM cells (**Figure 2**). When invading pathogenic bacteria are engulfed by macrophages, a series of antibacterial defense mechanisms are activated. The destruction of internalized microorganisms is the key to the innate immune response (2). Bony fish macrophages possess powerful bactericidal ability (18). Here, both LYC-FM and LYC-RM cells also exhibited bactericidal ability. Fish macrophages can destroy the invading pathogens by ROS and NO, and the quantity of ROS and NO production serves as an indicator of the innate immune response (38, 39, 47, 48). In teleost, IFN-γ can induce phagocytes to produce ROS and NO (49). In our previous study, IFN-γ was found to induce large yellow croaker primary macrophages to produce ROS and NO (31). In this study, both LYC-FM and LYC-RM cells produced the higher levels of ROS and NO in response to r*Lc*IFN-γ treatment compared to rTrx (**Figure 3**). These results were consistent with those observed in large yellow croaker primary macrophages (31). The ability of LYC-FM and LYC-RM cells to synthesize ROS and NO suggests that they may share the same effector molecules for microbicidal activities with primary macrophages.

Macrophages play an important role in antigen presentation process. In this process, CTSS can mediate proteolysis of endocytosed polypeptides (30). In the murine macrophage cell line RAW264.7, IFN-γ induces the expression of CTSS through direct binding of PU.1 to CTSS promoter (28). Our previous results showed that CTSS could be detected in large yellow croaker primary macrophages (30). However, the functions of PU.1 in regulating the expression of CTSS are unclear in this species. Here, PU.1 was found to exist in two transcripts, PU.1a and PU.1b, and both were constitutively expressed in LYC-FM and LYC-RM cells (**Figures 4**, **5**). In addition, their expression levels were upregulated by r*Lc*IFN-γ protein (**Figure 6A**). Additionally, r*Lc*IFN-γ, PU.1a, and PU.1b all could induce the expression of CTSS (**Figure 6**). These results were in accord with those in murine animals (22, 28). PU.1a or PU.1b could directly bind to the promoter region of CTSS and upregulate the expression of CTSS in large yellow croaker (**Figure 7**), but the binding regions of PU.1a and PU.1b to the CTSS promoter were somewhat different (**Figures 7**, **8**). This may reflect a difference between PU.1a and PU.1b in the regulation of CTSS expression as well as in the regulation of antigen presentation. Through the ChIP assay, we demonstrated that three Ets motifs within the CTSS promoter were recognized by PU.1a and all of four recognized by PU.1b. These results imply that PU.1b may competitively bind to the same motifs with PU.1a in the regulation of CTSS expression. It was notable that the first Ets motif in the CTSS promoter may be necessary for PU.1b regulation of CTSS expression (**Figures 7D, E**). PU.1a and PU.1b contain three different domains, and different domains have their own features. The three truncated mutants of PU.1a or PU.1b could not activate the CTSS promoter, suggesting that all three domains of PU.1a or PU.1b were essential for the initiation of CTSS expression (**Figure 9**).

In short, two macrophage cell lines (LYC-FM and LYC-RM) from large yellow croaker were established and characterized. Then we clarified the functions of transcription factors PU.1a and PU.1b in the regulation of CTSS expression by using these macrophages as a model system. Our results will be helpful for understanding the functions of PU.1 in bony fish. However, further studies are required to determine whether PU.1a or PU.1b cooperate with other transcription factors, such as IRF1 and IRF2, to regulate CTSS expression.

## Data Availability Statement

The raw data supporting the conclusions of this article will be made available by the authors, without undue reservation.

## Ethics Statement

The animal study was reviewed and approved by the Committee on the Ethics of Animal Experiments of the Fujian Agriculture and Forestry University.

## Author Contributions

X-YZ performed most of the experiments, analyzed the data, and wrote the manuscript. XZ and JC helped with the partial plasmid constructing. XW and KL help with experimental operations. XC designed the research and revised the manuscript. All authors contributed to the article and approved the submitted version.

## Funding

This work was supported by the National Natural Science Foundation of China (U1905204, 32102837), Natural Science Foundation of Fujian Province (2021J05021), grant from the Fujian Science and Technology Department (2021N5008), and National Training Program of Innovation and Entrepreneurship for Undergraduates (202110389010).

## Conflict of Interest

The authors declare that the research was conducted in the absence of any commercial or financial relationships that could be construed as a potential conflict of interest.

## Publisher’s Note

All claims expressed in this article are solely those of the authors and do not necessarily represent those of their affiliated organizations, or those of the publisher, the editors and the reviewers. Any product that may be evaluated in this article, or claim that may be made by its manufacturer, is not guaranteed or endorsed by the publisher.

## References

[1] Biswas SK Mantovani A. Macrophage Plasticity and Interaction With Lymphocyte Subsets: Cancer as a Paradigm. Nat Immunol (2010) 11:889–96. doi: 10.1038/ni.1937 20856220

[2] Hodgkinson JW Grayfer L Belosevic M. Biology of Bony Fish Macrophages. Biology (2015) 4:881–906. doi: 10.3390/biology4040881 26633534 PMC4690021

[3] Wynn TA Chawla A Pollard JW. Macrophage Biology in Development, Homeostasis and Disease. Nature (2013) 496:445–55. doi: 10.1038/nature12034 PMC3725458 23619691

[4] Braun-Nesje R Bertheussen K Kaplan G Seljelid R. Salmonid Macrophages: Separation, *In Vitro* Culture and Characterization. J Fish Dis (1981) 4:141–51. doi: 10.1111/j.1365-2761.1981.tb01118.x

[5] Sørensen K Sveinbjørnsson B Dalmo R Smedsrød B Bertheussen K. Isolation, Cultivation and Characterization of Head Kidney Macrophages From Atlantic Cod, *Gadus Morhua* L. J Fish Dis (1997) 20:93–107. doi: 10.1046/j.1365-2761.1997.d01-112.x

[6] Neumann NF Barreda DR Belosevic M. Generation and Functional Analysis of Distinct Macrophage Sub-Populations From Goldfish (*Carassius Auratus* L.) Kidney Leukocyte Cultures. Fish Shellfish Immunol (2000) 10:1–20. doi: 10.1006/fsim.1999.0221 10938719

[7] Bayne CJ. Pronephric Leucocytes of *Cyprinus Carpio*: Isolation, Separation and Characterization. Vet Immunol Immunop (1986) 12:141–51. doi: 10.1016/0165-2427(86)90118-2 3765337

[8] Weeks B Warinner J. Functional Evaluation of Macrophages in Fish From a Polluted Estuary. Vet Immunol Immunopathol (1986) 12:313–20. doi: 10.1016/0165-2427(86)90135-2 3765351

[9] Tahir A Secombes C. Modulation of Dab (*Limanda Limanda*, L.) Macrophage Respiratory Burst Activity. Fish Shellfish Immunol (1996) 6:135–46. doi: 10.1006/fsim.1996.0014

[10] Awasthi A Rathore G Pradhan P Rebello S Khan M Lakra W. Isolation and Characterization of Head Kidney Derived Macrophages of *Labeo Rohita* . J Environ Biol (2013) 35(5):949. doi: 10.1007/s11367-014-0772-8 25204072

[11] Rebello SC Rathore G Punia P Sood N Elangovan V. Development and Characterization of a Continuous Macrophage Cell Line, LRTM, Derived From Thymus of *Labeo Rohita* (Hamilton 1822). In Vitro Cell Dev-An (2014) 50:22–38. doi: 10.1007/s11626-013-9674-5 23955429

[12] Sha Z Wang L Sun L Chen Y Zheng Y Xin M. Isolation and Characterization of Monocyte/Macrophage From Peripheral Blood of Half Smooth Tongue Sole (*Cynoglossus Semilaevis*). Fish Shellfish Immunol (2017) 65:256–66. doi: 10.1016/j.fsi.2017.04.015 28433719

[13] Li Q-F Ai Q-H Mai K-S Zheng Y-F Xu W Zhang W-B. Development of a Short-Term *In Vitro* Model of Macrophages From Large Yellow Croaker, *Larmichthys Crocea* (Richardson, 1846). Acta Hydrobiol Gica Sinica (2013) 37:885–91. doi: 10.7541/2013.113

[14] Ganassin RC Bols NC. Development of a Monocyte/Macrophage-Like Cell Line, RTS11, From Rainbow Trout Spleen. Fish Shellfish Immunol (1998) 8:457–76. doi: 10.1006/fsim.1998.0153

[15] Wang R Neumann N Shen Q Belosevic M. Establishment and Characterization of a Macrophage Cell Line From the Goldfish. Fish Shellfish Immunol (1995) 5:329–46. doi: 10.1006/fsim.1995.0032

[16] Chaudhary DK Sood N Pradhan P Singh A Punia P Agarwal N. Establishment of a Macrophage Cell Line From Adherent Peripheral Blood Mononuclear Cells of *Catla Catla* . In Vitro Cell Dev-An (2012) 48:340–8. doi: 10.1007/s11626-012-9516-x 22678754

[17] Awasthi A Rathore G Sood N Khan M Lakra W. Establishment of a Leukocyte Cell Line Derived From Peritoneal Macrophages of Fish, *Labeo Rohita* (Hamilton, 1822). Cytotechnology (2015) 67:85–96. doi: 10.1007/s10616-013-9660-5 24248274 PMC4294834

[18] Cui K Li Q Xu D Zhang J Gao S Xu W. Establishment and Characterization of Two Head Kidney Macrophage Cell Lines From Large Yellow Croaker (*Larimichthys Crocea*). Dev Comp Immunol (2020) 102:103477. doi: 10.1016/j.dci.2019.103477 31470020

[19] Braun-Nesje R Kaplan G Seljelid R. Rainbow Trout Macrophages *In Vitro*: Morphology and Phagocytic Activity. Dev Comp Immunol (1982) 6:281–91. doi: 10.1016/S0145-305X(82)80011-6 7095232

[20] Anderson KL Smith KA Conners K McKercher SR Maki RA Torbett BE. Myeloid Development Is Selectively Disrupted in PU. 1 Null Mice. Blood (1998) 91:3702–10. doi: 10.1182/blood.V91.10.3702 9573007

[21] Ghisletti S Barozzi I Mietton F Polletti S De Santa F Venturini E. Identification and Characterization of Enhancers Controlling the Inflammatory Gene Expression Program in Macrophages. Immunity (2010) 32:317–28. doi: 10.1016/j.immuni.2010.02.008 20206554

[22] DeKoter RP Lee H-J Singh H. PU. 1 Regulates Expression of the Interleukin-7 Receptor in Lymphoid Progenitors. Immunity (2002) 16:297–309. doi: 10.1016/S1074-7613(02)00269-8 11869689

[23] Celada A Borràs FE Soler C Lloberas J Klemsz M van Beveren C. The Transcription Factor PU. 1 Is Involved in Macrophage Proliferation. J Exp Med (1996) 184:61–9. doi: 10.1084/jem.184.1.61 PMC2192661 8691150

[24] Karpurapu M Wang X Deng J Park H Xiao L Sadikot RT. Functional PU. 1 in Macrophages has a Pivotal Role in NF-κb Activation and Neutrophilic Lung Inflammation During Endotoxemia. Blood (2011) 118:5255–66. doi: 10.1182/blood-2011-03-341123 PMC3217408 21937699

[25] Rechsteiner M Rogers SW. PEST Sequences and Regulation by Proteolysis. Trends Biochem Sci (1996) 21:267–71. doi: 10.1016/S0968-0004(96)10031-1 8755249

[26] Marecki S Fenton MJ. PU. 1/Interferon Regulatory Factor Interactions. Cell Biochem Biophys (2000) 33:127–48. doi: 10.1385/CBB:33:2:127 11325034

[27] Hsieh C-S DeRoos P Honey K Beers C Rudensky AY. A Role for Cathepsin L and Cathepsin S in Peptide Generation for MHC Class II Presentation. J Immunol (2002) 168:2618–25. doi: 10.4049/jimmunol.168.6.2618 11884425

[28] Layne MD Ye Q Le L Baron RM Perrella MA Santambrogio L. IFN Regulatory Factor-1 Regulates IFN-γ-Dependent Cathepsin S Expression. J Immunol (2002) 168:4488–94. doi: 10.4049/jimmunol.168.9.4488 11970993

[29] Wang Y Baron RM Zhu G Joo M Christman JW Silverman ES. PU. 1 Regulates Cathepsin S Expression in Professional APCs. J Immunol (2006) 176:275–83. doi: 10.4049/jimmunol.176.1.275 16365419

[30] Li Q Ao J Mu Y Yang Z Li T Zhang X. Cathepsin S, But Not Cathepsin L, Participates in the MHC Class II-Associated Invariant Chain Processing in Large Yellow Croaker (*Larimichthys Crocea*). Fish Shellfish Immunol (2015) 47:743–50. doi: 10.1016/j.fsi.2015.10.009 26475363

[31] Yang S Li Q Mu Y Ao J Chen X. Functional Activities of Interferon Gamma in Large Yellow Croaker *Larimichthys Crocea* . Fish Shellfish Immunol (2017) 70:545–52. doi: 10.1016/j.fsi.2017.09.051 28939528

[32] Li C Wang S Ren Q He T Chen X. An Outbreak of Visceral White Nodules Disease Caused by Pseudomonas Plecoglossicida at a Water Temperature of 12°C in Cultured Large Yellow Croaker (*Larimichthys Crocea*) in China. J Fish Dis (2020) 43:1353–61. doi: 10.1111/jfd.13206 32851664

[33] Mu Y Huo J Guan Y Fan D Xiao X Wei J. An Improved Genome Assembly for *Larimichthys Crocea* Reveals Hepcidin Gene Expansion With Diversified Regulation and Function. Commun Biol (2018) 1:195. doi: 10.1038/s42003-018-0207-3 30480097 PMC6240063

[34] Ao J Mu Y Xiang L-X Fan D Feng M Zhang S. Genome Sequencing of the Perciform Fish *Larimichthys Crocea* Provides Insights Into Molecular and Genetic Mechanisms of Stress Adaptation. PloS Genet (2015) 11:e1005118. doi: 10.1371/journal.pgen.1005118 25835551 PMC4383535

[35] Zhang X-Y Cui Z-W Zhou Y-Y Chen D-D Zhang Y-A. Neutrophil Functions Can be Regulated by IL-35, Which Is Mainly Expressed in IL-15rα^+^ Cells in Grass Carp (*Ctenopharyngodon Idella*). Dev Comp Immunol (2021) 121:104103. doi: 10.1016/j.dci.2021.104103 33857470

[36] Awasthi A Rathore G Pradhan P Rebello S Khan M Lakra W. Isolation and Characterization of Head Kidney Derived Macrophages of *Labeo Rohita* . J Environ Biol (2014) 35:949. doi: 10.1007/s11367-014-0772-8 25204072

[37] Mao K Chen W Mu Y Ao J Chen X. Identification of Two IL-4/13 Homologues in Large Yellow Croaker (*Larimichthys Crocea*) Revealed Their Similar Roles in Inducing Alternative Activation of Monocytes/Macrophages. Fish Shellfish Immunol (2018) 80:180–90. doi: 10.1016/j.fsi.2018.06.002 29870826

[38] Datta S Ghosh D Saha DR Bhattacharaya S Mazumder S. Chronic Exposure to Low Concentration of Arsenic Is Immunotoxic to Fish: Role of Head Kidney Macrophages as Biomarkers of Arsenic Toxicity to Clarias Batrachus. Aquat Toxicol (2009) 92:86–94. doi: 10.1016/j.aquatox.2009.01.002 19237206

[39] Hermann AC Kim CH. Effects of Arsenic on Zebrafish Innate Immune System. Mar Biotechnol (2005) 7:494–505. doi: 10.1007/s10126-004-4109-7 16007375

[40] Wang X Wang K Nie P Chen X Ao J. Establishment and Characterization of a Head Kidney Cell Line From Large Yellow Croaker *Pseudosciaena Crocea* . J Fish Biol (2014) 84:1551–61. doi: 10.1111/jfb.12386 24773544

[41] Sinder BP Pettit AR McCauley LK. Macrophages: Their Emerging Roles in Bone. J Bone Miner Res (2015) 30:2140–9. doi: 10.1002/jbmr.2735 PMC4876707 26531055

[42] Takizawa F Magadan S Parra D Xu Z Korytář T Boudinot P. Novel Teleost CD4-Bearing Cell Populations Provide Insights Into the Evolutionary Origins and Primordial Roles of CD4^+^ Lymphocytes and CD4^+^ Macrophages. J Immunol (2016) 196:4522–35. doi: 10.4049/jimmunol.1600222 PMC5100900 27183628

[43] Lu X-J Chen Q Rong Y-J Chen F Chen J. CXCR3. 1 and CXCR3. 2 Differentially Contribute to Macrophage Polarization in Teleost Fish. J Immunol (2017) 198:4692–706. doi: 10.4049/jimmunol.1700101 28500070

[44] Lugo-Villarino G Balla KM Stachura DL Bañuelos K Werneck MB Traver D. Identification of Dendritic Antigen-Presenting Cells in the Zebrafish. P Natl Acad Sci USA (2010) 107:15850–5. doi: 10.2307/27862346 PMC2936643 20733076

[45] Cerezuela R Cuesta A Meseguer J Esteban MÁ. Effects of Dietary Vitamin D3 Administration on Innate Immune Parameters of Seabream (*Sparus Aurata* L.). Fish Shellfish Immunol (2009) 26:243–8. doi: 10.1016/j.fsi.2008.11.004 19061960

[46] Joerink M Ribeiro CM Stet RJ Hermsen T Savelkoul HF Wiegertjes GF. Head Kidney-Derived Macrophages of Common Carp (*Cyprinus Carpio* L.) Show Plasticity and Functional Polarization Upon Differential Stimulation. J Immunol (2006) 177:61–9. doi: 10.4049/jimmunol.177.1.61 16785499

[47] Forlenza M Nakao M Wibowo I Joerink M Arts JA Savelkoul HF. Nitric Oxide Hinders Antibody Clearance From the Surface of Trypanoplasma Borreli and Increases Susceptibility to Complement-Mediated Lysis. Mol Immunol (2009) 46:3188–97. doi: 10.1016/j.molimm.2009.08.011 19735943

[48] Hanington PC Belosevic M. Interleukin-6 Family Cytokine M17 Induces Differentiation and Nitric Oxide Response of Goldfish (*Carassius Auratus* L.) Macrophages. Dev Comp Immunol (2007) 31:817–29. doi: 10.1016/j.dci.2006.12.001 17250891

[49] Gan Z Chen SN Huang B Zou J Nie P. Fish Type I and Type II Interferons: Composition, Receptor Usage, Production and Function. Rev Aquacult (2020) 12:773–804. doi: 10.1111/raq.12349

