# Supplementary material for: PU.1 Regulates Cathepsin S Expression in Large Yellow Croaker (Larimichthys crocea) Macrophages
Source: Front Immunol. 2022 Jan 5;12:819029. doi: 10.3389/fimmu.2021.819029 (PMC8766968; doi:10.3389/fimmu.2021.819029)
Supplement: Supplementary file 1 [file DataSheet_1.docx]

***Supplementary Material***

**Supplementary Table**

**Table S1 Primers used in this study**

| Gene  (Accession No.) | Primer | Sequences (5’-3’) | Application |
| --- | --- | --- | --- |
| PU.1  (XM_010748147,  XM_010748148)  F4/80  (XM_027283123)  CD11b  (XM_027289242)  CD68  (NM_001319937)  MCSFRa  (XM_010741048)  MCSFRb  (XM_010731461)  MPEG1  (XM_019259929)  LYZ  (XM_019258614)  CTSS  (KF314187)  β-Actin  (XM_027284923)  CTSS  (NC_040023.1) | PU1-F  PU1-R  PU1a-qF  PU1b-qF  PU1-qR  PU1-F1  PU1-R1  PU1-F2  PU1-R2  PU1-F3  PU1-R3  F4/80-F  F4/80-R  CD11b-F  CD11b-R  CD68-F  CD68-R  MCSFRa-F  MCSFRa-R  MCSFRb-F  MCSFRb-R  MPEG1-F  MPEG1-R  LYZ-F  LYZ-R  CTSS-qF CTSS-qR  β-Actin-F  β-Actin-R  CTSS-pF  CTSS-pR  CTSS-pF1  CTSS-pR1  CTSS-pF2  CTSS-pR2  CTSS-pF3  CTSS-pR3  CTSS-pF4  CTSS-pR4  CTSS-pF5  CTSS-pR5  CTSS-pF6  CTSS-pR6  CTSS-pF7  CTSS-pR7 | GATTCTCATGCTGCCTTGTTTAC  GATTTCAGCAAAATAAATGACAGC  CTTCCACTCTGAGCGTGTCCA  GAGGAGATTTATGAAGAGCTTCA  CATCCTCTCCATCTGATACTTC  GACGACAAGCTTGCGGCCGCGATGGACGGATATGTCATATCACC  TAGATGATCTATCGATGAATTTCAGTGAGGATACTGACGTAAGT  GACGACAAGCTTGCGGCCGCGATGGACGGACTCGGACCTCTCTCCGTGTC  TAGTGTCTTTCCTGAAGGAGGAGGTCGGGGGGTAATATGGGA  TCCCATATTACCCCCCGACCTCCTCCTTCAGGAAAGACACTA  TAGATGATCTATCGATGAATTTCAGTGAGGATACTGACGTAAGTAGAGGTTCCTCAGGACTGCCTCACTCTTCTTGTTGTTAGTGTCTTTCC  CATACTGGGTTTGATGTGGGTG  CCGTGTACCTTGTGACTGAGTA  CCCAAACAACCGAGCTTACATC  CCACTTTCAGATTATCCACACAA  GAGTACTCTGCCAACAACCAGT  GGCAGCACAGACGTCAGATGT  CCATCAACGTCATTCAGAAACTT  GTTTCCTGTGTCGGTAAGGTCT  CTGGTGGTTAGCTGTAAGATCT  TTGACATAGTGTTGGCTCATAGT  GGTATCTGTTTGGAGGCCTATA  ATGGTACTGAGAATCTGGTGGC  CAGCTGAGCTCCTGAACTATAA  CCCATTCACAACGCTGGTAGA  GCCCAGATTTGCGTTCTACAG  CCCTCTGCAGCTACATGATGG  CATGGACTCCGGTGATGGTGT  ACAGCTTCTCCTTGATGTCACG  GATAGTGACACTTTATTTGGTATTC  CACTCTGCTCTTATTAATGTGTC  CAAGACATGTGCAGTGTTAAATCTTCTCCTGTTTTGTTGCTTCATCCAATAA  TTATTGGATGAAGCAACAAAACAGGAGAAGATTTAACACTGCACATGTCTTG  CAGCAGCAGCAGCCTCAGGAGAGAATTGTCAAATTTGCACACAGAATAT  ATATTCTGTGTGCAAATTTGACAATTCTCTCCTGAGGCTGCTGCTGCTG  GCAAAATGCAACGTCAGAATCTATTCTCAATCATCTGACGATGATTCAGCT  AGCTGAATCATCGTCAGATGATTGAGAATAGATTCTGACGTTGCATTTTGC  AATAAACCTGATCTGATGCAGAAATCTCGCTTGTCGTCCAAACAGTATTG  CAATACTGTTTGGACGACAAGCGAGATTTCTGCATCAGATCAGGTTTATT  TGCATTTCACATCACAGCCTG  GAGAAAATCATCTGAGAACATGG  CCTCCAATTTGATCCAGAACCA  CAGCAGGGAGGATGAACATGTT  TCGTGGACTGCAGAAGAAGGTT  GCCAGATGTTGTTGAGGTCACT | cDNA amplification  Real-time PCR  Eukaryotic expression  Mutant construction  Gene expression detection  Gene expression detection  Gene expression detection  Gene expression detection  Gene expression detection  Gene expression detection  Gene expression detection  Real-time PCR  Real-time PCR  Promoter amplification  Mutation of promoter  Mutation of promoter  Mutation of promoter  Mutation of promoter  ChIP assay |

**Supplementary Figures**


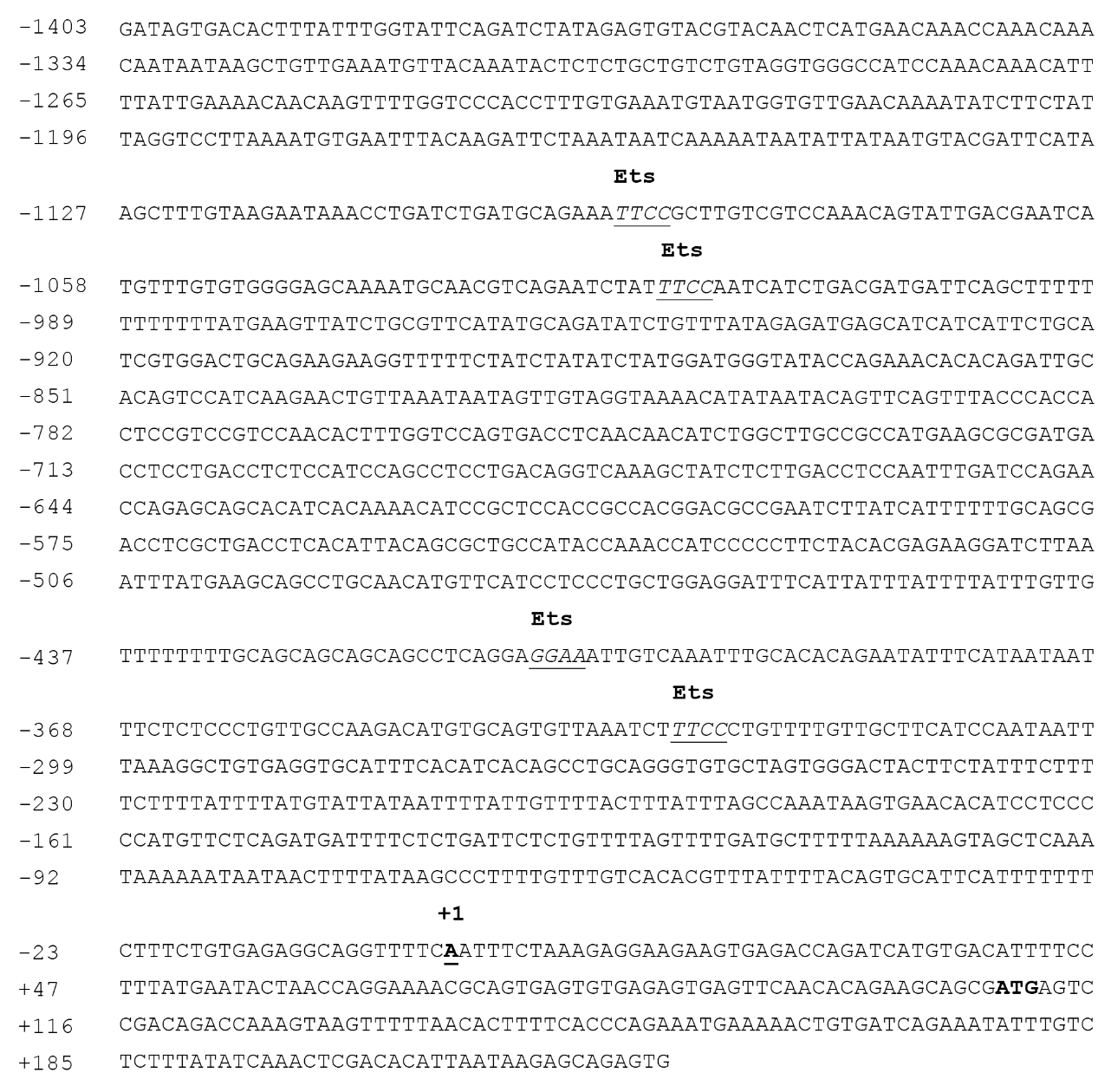


**Figure S1. Prediction of the Ets motifs in the promoter region of large yellow croaker CTSS.** The putative Ets motifs were predicated. The transcriptional start site was designated by +1.
